# Pattern of Fractures Among Road Traffic Accident Victims Requiring Hospitalization: Single-institution Experience in Saudi Arabia

**DOI:** 10.7759/cureus.6550

**Published:** 2020-01-03

**Authors:** Abdulrahman A Aloudah, Faisal A Almesned, Abdullah A Alkanan, Turki Alharbi

**Affiliations:** 1 Orthopedics, Buraidah Central Hospital, Buraidah, SAU; 2 Family Medicine, Qassim Medical College, Buraidah, SAU

**Keywords:** fractures, orthopedic, road traffic accident, incidence

## Abstract

Introduction

A bone fracture is a medical condition in which there is a partial or complete break in the continuity of the bone. One of the factors causing bone fractures is Road Traffic Accidents (RTAs), which is the first leading cause of death among people aged 15-29 years. This study aimed to determine the incidence of bone fractures among RTAs in Buraidah Central Hospital and to identify associations between gender, age, and fractures.

Materials and methods

This is a retrospective study and review of patients’ medical records in Buraidah Central Hospital, of those who had an RTA in 2018 and 2019. The information taken included age, gender, and type of fracture. All patients with RTAs in the period between January 2018 and October 2019 were recruited and data were collected from patients’ medical records. The data analysis was carried out using Statistical Packages for Software Sciences (SPSS) version 21 (SPSS, Chicago, IL, USA).

Results

There were 301 incidences of RTAs with fractured bones presented at Buraidah Central Hospital during the study period. Bilateral or multiple was the most commonly known sites of fractures. We also observed that the most commonly fractured bone among males was the femur (28.2%) while a humerus fracture was the most common among females (20.8%). Males were significantly higher in the younger age group (p<0.001). Additionally, the radius shaft was more associated with the younger age group (p=0.043) while the femur was more associated with the oldest age group (p=0.013).

Conclusion

Femur fractures were the most commonly known fractures among males while a humerus fracture was the most common fracture among females. In males, a fractured radius shaft and a fractured femur were the factors most associated with the age group.

## Introduction

According to the World Health Organization (WHO), road traffic accidents (RTAs) are the first leading cause of death among people aged 15-29 [1]. It is also predicted that motor vehicle accident injuries would be the sixth commonest leading cause of death by the year 2020 and the fifth leading cause by 2030 [2-3]. In Saudi Arabia, road traffic accidents are considered a major health hazard leading to injuries and death; between 1971 and 1997, 564,762 people were injured or died in a motor vehicle accident in Saudi Arabia [4]. Recent statistics from the Ministry of Interior - General Directorate of Traffic show that the total number of traffic accidents is 352,464, which reflects a dangerous impact on the patient's health and economic status [5]. In this study, we aimed to find the incidence of bone fractures among RTA patients in Buraidah Central Hospital (BCH), identify the types of fractures, and identify associations between gender and age.

## Materials and methods

This study is a single-institution study conducted at Buraidah Central Hospital in Qassim, Saudi Arabia. It is a retrospective study and reviews the medical records of patients who were admitted to the hospital and had a fracture following an RTA in the period between January 2018 and October 2019. The information collected includes demographic data (age and gender), type of fracture, and mode of injury. The data collected were analyzed using descriptive statistical measures such as frequency distribution and percentages. The comparison between gender, fractured bones, and site of fracture among age groups has been conducted using the chi-square test. A p-value of <0.05 has been considered statistically significant. All data analyses were carried out using Statistical Packages for Software Sciences (SPSS) version 21 (SPSS, Chicago, IL, USA).

## Results

There were 301 orthopedic fracture bone cases enrolled in this study. Table 1 presents the baseline characteristics of the patients. The age range was from two to 90 years (mean 31.6) of whom the majority were in the middle-age group (21-30 years) with 36.5%, followed by the 10-20 years' group (20.3%). It was a male-dominant cohort (82.4% vs 17.6%). With regards to the sites of fractures, the majority suffered fractures from bilateral or multiple sites (35.2%), followed by the right side (24.6%) and the left side (23.6) while the least were from the axial part (16.6%).

**Table 1:** Baseline characteristics of patients

Study variables	Overall N (%) (n=301)
Age group in years	
<10 years	09 (03.0%)
10 – 20 years	61 (20.3%)
21 – 30 years	110 (36.5%)
41 – 50 years	56 (18.6%)
>50 years	43 (14.3%)
Gender	
Male	248 (82.4%)
Female	53 (17.6%)
Site of fractures	
Right	74 (24.6%)
Left	71 (23.6%)
Bilateral or multiple	106 (35.2%)
Axial	50 (16.6%)

**Figure 1:**
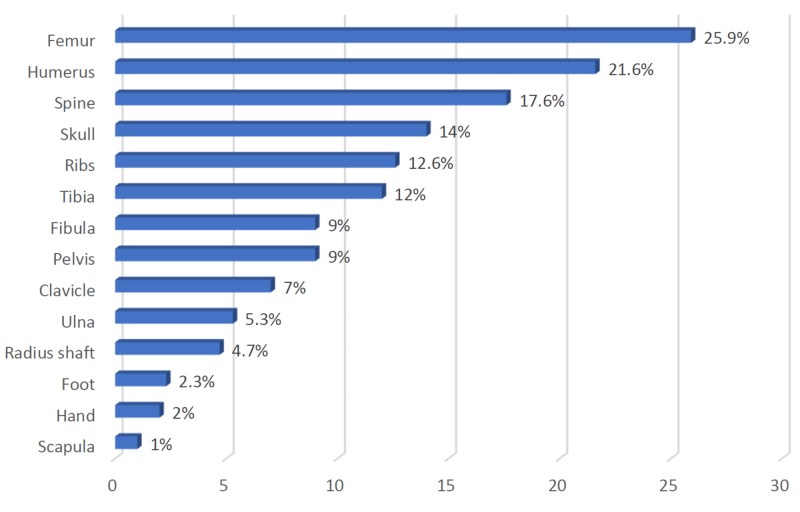
Distribution of patients' fractured bone

The most commonly fractured bone was the femur (25.9%), followed by the humerus (21.6%) and spine (17.6%), while the least common was the scapula (1%) (Figure 1).

**Figure 2:**
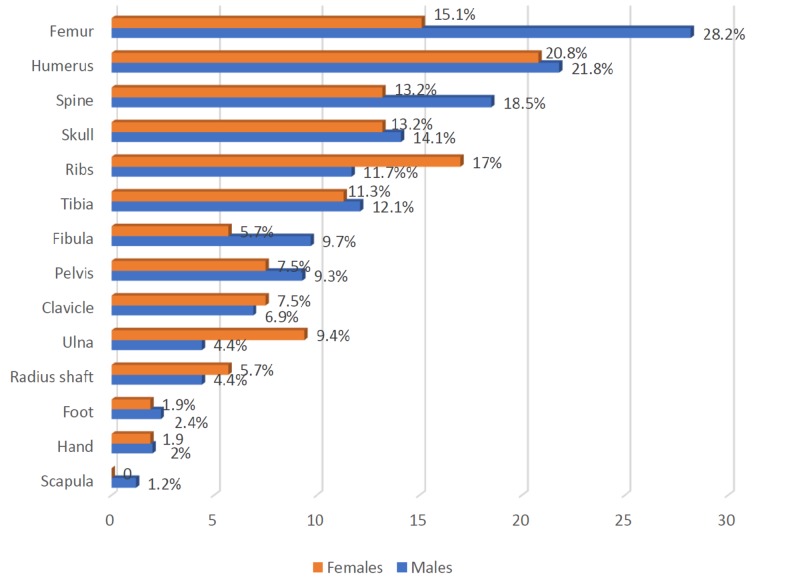
Comparison of fractured bones among males and females

Figure 2 depicts the fractured bones of males and females. Based on the results, the most commonly fractured bones in males was the femur (28.2%), followed by the humerus (21.8%), while the least common was the scapula (1.2%). In females, the most commonly fractured bone was also the humerus (20.8%), followed by the ribs (17%), while the least common were the hand and foot (both 1.9%).

When measuring the relationship between the age group in years among gender, fractured bones, and sites of fractures, we found that gender has a significant relationship with the age group (p<0.001). A fractured radius shaft was also statistically significant to age group (p=0.043) where the younger age group (≤25 years) was more associated with a fractured radius shaft. We also found out that a femur fracture had a significant association with age group (p=0.013) where age group 25 years or less is significantly higher for having a femur fracture. On the other hand, sites of fractures and other fractured bones, such as skull, clavicle, ribs humerus, ulna, tibia, fibula, pelvis, spine, scapula, foot, and hand, were not statistically significant for age groups (Table 2).

**Table 2:** Relationship between gender, fractured bones, and sites of fractures among age groups (n=301)

Study variables	Age group			P-value ^§^
	≤25 years N (%) ^(n=128)^	26 – 49 years N (%) ^(n=127)^	≥50 years N (%) ^(n=46)^	
Gender				
Male	113 (88.3%)	108 (85.0%)	27 (58.7%)	<0.001 **
Female	15 (11.7%)	19 (15.0%)	19 (41.3%)	
Fractured bones				
Skull	17 (13.3%)	20 (15.7%)	05 (10.9%)	0.711
Clavicle	11 (08.6%)	07 (05.5%)	03 (06.5%)	0.652
Ribs	14 (10.9%)	19 (15.0%)	05 (10.9%)	0.614
Humerus	27 (21.1%)	29 (22.8%)	09 (19.6%)	0.862
Radius shaft	10 (07.8%)	04 (03.1%)	0	0.043 **
Ulna	11 (08.6%)	05 (03.9%)	0	0.052
Femur	41 (32.0%)	22 (17.3%)	15 (32.6%)	0.013 **
Tibia	16 (12.5%)	17 (13.4%)	03 (06.5%)	0.517
Fibula	15 (11.7%)	08 (06.3%)	04 (08.7%)	0.314
Pelvis	07 (05.5%)	16 (12.6%)	04 (08.7%)	0.201
Spine	21 (16.4%)	22 (17.3%)	10 (21.7%)	0.662
Foot	03 (02.3%)	04 (03.1%)	0	0.599
Hand	01 (0.80%)	04 (03.1%)	0	0.325
Scapula	02 (01.6%)	01 (0.80%)	0	0.834
Site of Fractures				
Right	33 (25.8%)	31 (24.4%)	10 (21.7%)	0.205
Left	32 (25.0%)	26 (20.5%)	13 (28.3%)	
Bilateral or multiple	50 (39.1%)	43 (33.9%)	13 (28.3%)	
Axial	13 (10.2%)	27 (21.3%)	10 (21.7%)	

## Discussion

This study sought to determine the incidence of bone fractures among RTA cases at Buraidah Central Hospital. In this study, a total of 301 cases of RTAs were determined in a two-year period (years 2018-2019) and the majority of the victims were in the 21-30-year-old group (36.5%). This report is in accordance with a paper published by Sonbol et al. [6]. They documented that among 591 patients, half (50.4%) were in the 16 to <30-year-old group. This report has been further validated by Kumar [7]. Based on his findings, most victims were young, in the age group of 15-30 years (33.9%), which was consistent with our study outcome. On the other hand, Sadat-Ali et al. [8], when evaluating the epidemiology of fractures and dislocations in urban communities among 1428 recruited patients, found 584 of the patients were victims of RTAs, which was relatively higher than the incidence of RTA in the present study, although the previous study was conducted over a five-year period as compared to our study, which was conducted over an approximately two-year period only. They also reported that the average age of males was 28.9 while the average age of females was 41.6; among them, 40.9% of males were below 40 years old, which was also congruent with our report. Another published study in India [9] reported that the most common age group among 4041 patients who presented with fractures and dislocation was the fourth-decade group followed by the third-decade group, which was not consistent with our study findings.

Males were generally more victims of RTAs than females. In our study, 82.4% of the males were involved in RTAs, which was consistent with the study of Sonbol et al. [6]. They reported that among 591 patients, 78% were males. This has been further supported by Kumar [7], where he accounted that 79.9% of the RTA victims were males, which generally means that RTAs are more associated with males as compared to females.

Moreover, we documented that the most common sites of fractures among RTA victims were bilateral or multiple sites, followed by the right or left side. For femoral shaft fractures, the most common site of fracture was the head and chest which was reported by Sonbol et al [6]. Our report coincided with the paper published by Kumar [7]. He observed that multiple fractures in which more than two sites were involved were found in the majority of patients (21.4%). Another published paper in Saudi Arabia reported that the lower limb and upper limb were the most common site of fracture among patients admitted in the orthopedic ward [8]. This has been supported by Meena et al. [10], where they documented that upper limb fractures were recorded more than lower limb fractures, whereas Pan et al. revealed that fracture of the upper limb, fracture of lower limb, and fracture of the spine and trunk were the most commonly known sites of fracture [9].

In this study, we observed that femur fractures were the most frequently found fractures among men while humerus fractures were the most frequently found among women. Fractures suffered in RTAs differ with each region. For example, Sonbol et al. [6] reported that tibial and patellar fractures were found among males, and a higher percentage of associated distal femoral fractures were found among female patients. They further observed that fractures of the neck of femur and tibia were more among patients aged from 30 to <60 years while distal femoral fractures were more among patients in the 60 years and more group. In India [7], when they assessed the most commonly dislocated joint being suffered by RTA victims, they found that the wrist joint was the most commonly dislocated joint followed by elbow dislocation, while in Taiwan [9], in a span of one decade, they documented that fractures of the skull, intracranial injury, and contusion with intact skin surface were the most common injury patterns among RTA victims.

Finally, when we assessed which factors are more associated with age group, we observed that being a male, a fractured radius shaft and a fractured femur are the factors being concomitant with the age group. In the western region [6], they reported that the tibia and neck were the significant factors for age group, which was not consistent with our study findings, while in Taiwan [9], they reported a significant relation of male gender, older age, low income, and admission to a high-level hospital to the observed fracture patterns, which was incongruent with our study results.

## Conclusions

We observed 301 cases of RTAs in a two-year period. Femur fractures were the most commonly known fractures among males while humerus fractures were the most common fractures among females. In males, fractured radius shaft and fractured femur were the factors associated with age group. RTAs account for many orthopedic fractures that cause long hospitalization and increased mortality rates. In this regard, an awareness campaign at the maximum level is necessary for targeting the high-risk group, which is adult males. Furthermore, good maintenance of roads, as well as proper road signs, are also necessary to prevent the occurrence of RTAs. Finally, collective efforts within the government agencies to educate drivers on safe driving are vital for decreasing the high prevalence of RTAs in our region.
